# Tetrahydrobiopterin (BH_4_) Supplementation Prevents the Cardiorenal Effects of Diabetes in Mice by Reducing Oxidative Stress, Inflammation and Fibrosis

**DOI:** 10.3390/biomedicines10102479

**Published:** 2022-10-04

**Authors:** Ulises Novoa, Karen Soto, Cristian Valdés, Jorge Villaseñor, Adriana V. Treuer, Daniel R. González

**Affiliations:** 1 Departamento de Ciencias Básicas Biomédicas, Facultad de Ciencias de la Salud, Universidad de Talca, Avenida Lircay s/n, Talca 3460000, Chile; 2 Centro de Investigación de Estudios Avanzados del Maule (CIEAM), Vicerrectoría de Investigación y Postgrado, Universidad Católica del Maule, Talca 3466706, Chile; 3 Instituto de Química de Recursos Naturales, Universidad de Talca, Talca 3460000, Chile; 4 Departamento de Biología y Química, Facultad de Ciencias Básicas, Universidad Catolica del Maule, Talca 3466706, Chile

**Keywords:** nitric oxide synthase uncoupling, sapropterin, diabetic nephropathy

## Abstract

Background: The effects of diabetes on the cardiovascular system as well as in the kidney are profound, which include hypertrophy and fibrosis. Diabetes also induces oxidative stress, at least in part due to the uncoupling of nitric oxide synthase (NOS); this is a shift in NO production toward superoxide production due to reduced levels of the NOS cofactor tetrahydrobiopterin (BH_4_). With this in mind, we tested the hypothesis that BH_4_ supplementation may prevent the development of diabetic cardiomyopathy and nephropathy. Methods: Diabetes was induced in Balb/c mice with streptozotocin. Then, diabetic mice were divided into two groups: one group provided with BH_4_ (sapropterin) in drinking water (daily doses of 15 mg/kg/day, during eight weeks) and the other that received only water. A third group of normoglycemic mice that received only water were used as the control. Results: Cardiac levels of BH_4_ were increased in mice treated with BH_4_ (*p* = 0.0019). Diabetes induced cardiac hypertrophy, which was prevented in the group that received BH_4_ (*p* < 0.05). In addition, hypertrophy was evaluated as cardiomyocyte cross-sectional area. This was reduced in diabetic mice that received BH_4_ (*p* = 0.0012). Diabetes induced cardiac interstitial fibrosis that was reduced in mice that received BH_4_ treatment (*p* < 0.05). We also evaluated in the kidney the impact of BH_4_ treatment on glomerular morphology. Diabetes induced glomerular hypertrophy compared with normoglycemic mice and was prevented by BH_4_ treatment. In addition, diabetic mice presented glomerular fibrosis, which was prevented in mice that received BH_4_. Conclusions: These results suggest that chronic treatment with BH_4_ in mice ameliorates the cardiorenal effects of diabetes,_,_ probably by restoring the nitroso–redox balance. This offers a possible new alternative to explore a BH_4_-based treatment for the organ damage caused by diabetes.

## 1. Introduction

Diabetes mellitus is one of the most common chronic diseases worldwide, and continues to increase in numbers and significance, with characteristics of an epidemic, as modern lifestyles lead to reduced physical activity and increased obesity [1]. Diabetic cardiomyopathy is the manifestation in the myocardium of the alterations produced by the altered homeostasis of glucose metabolism, independent of coronary artery disease [2]. This cardiomyopathy is initially characterized by diastolic dysfunction and cardiac hypertrophy, with preserved ejection fraction. As diabetes progresses, systolic dysfunction and reduced ejection fraction develop. This process of cardiac deterioration in diabetes includes altered metabolism, inflammation, and oxidative stress, which result in apoptosis and fibrosis that further deteriorate the myocardium [3,4,5,6]. In addition, altered calcium handling was characterized in the diabetic cardiac myocytes. The reduced capacity of the sarcoplasmic reticulum Ca^2+^ pump SERCA2 results in a diminished storage capacity of Ca^2+^, which impairs cardiac contractility. Importantly, it also alters cardiac relaxation, which is evidenced in the diastolic dysfunction [7].

Diabetic nephropathy is another of the main complications of diabetes. In advanced stages, it is characterized by urinary albumin excretion [8]. It begins with a series of cellular and molecular changes that lead to morphological alterations, first in the glomerulus, then, in more advanced stages, in the tubules and interstitial space [8]. Glomeruli undergo hypertrophy, with a thickening of the basal membrane and basal tubular membrane, with a progressive accumulation of extracellular matrix components [9]. These ultrastructure changes are responsible for the functional alterations observed in diabetic nephropathy, such as proteinuria, hypertension, and, finally, renal failure. After hyperglycemia is chronically established, oxidative stress is one the main biochemical alterations that occur in the kidney [10,11], leading to a proinflammatory state [11].

Current treatments for the cardiorenal complications of diabetes are based on the control of blood glucose levels, mainly with metformin and sulfonylureas in type 2 diabetes mellitus and insulin mainly in type 1 diabetes [12]. More recently, clinical trials evaluating the organ target damage such as cardiac and renal complications with the use of di-peptidyl peptidase-4 (DPP4) inhibitors, glucagon-like peptide (GLP1) receptor agonists, and sodium-glucose co-transporter 2 (SGLT2) inhibitors have shown promising results [13,14,15,16]. At the preclinical level, pharmacological strategies are now directly focusing on end-organ damage processes such as fibrosis, inflammation, and oxidative stress [3].

In diabetes, several sources may contribute to the observed oxidative stress, such as xanthine oxidoreductase, nicotinamide adenine dinucleotide phosphate (NADPH) oxidases, mitochondria, and uncoupled nitric oxide synthases (NOS) [17]. A direct consequence of the increased production of reactive oxygen species (ROS) is the uncoupling of nitric oxide synthase [18]. This is due to the oxidation of tetrahydrobiopterin (BH_4_), an essential cofactor for NOS activity. When NOS is uncoupled, its activity is redirected toward the production of superoxide, instead of NO, further contributing to the oxidative process [19]. Because BH_4_ oxidation may also occur in oxidative states in the kidney, this leads to endothelial NOS uncoupling, which generates endothelial dysfunction in the kidney vasculature, including the glomerular capillaries, and afferent and efferent arterioles [20].

We tested the hypothesis that in diabetes, tetrahydrobiopterin supplementation leads to the recoupling of nitric oxide synthase 1 (NOS1), preventing cardiac remodeling and the advance of diabetic nephropathy, two of the main complications of chronic diabetes. 

These findings in the diabetic heart and kidney represent a potential translational tool with therapeutic value. The proposed investigation may have a translational impact and contribute to the basic knowledge of NOS uncoupling in the setting of diabetic cardiomyopathy.

## 2. Methods

### 2.1. Experimental Model and Protocol

Diabetes was induced in Balb/c mice (*n* = 30, male, 30–40 g) with the intraperitoneal injection of three doses (100, 100, and 200 mg/kg) of streptozotocin (Sigma, St. Louis, MO, USA) in 10 mM citrate buffer, pH 4.5. The control group received an injection of citrate. Then, diabetic mice were divided into two groups: one group provided with BH_4_ (sapropterin, Inpheno, InnoPharmax, Inc., Taipei City, Taiwan, Lot # 6P001) in drinking water (daily doses of 15 mg/kg/day, during eight weeks), and the other received only water. A third group of normoglycemic mice that received only water were used as the control. The protocol was approved by the Bioethics Committee of Universidad de Talca (# 2015-087-DG). Mice were kept in the animal facility of the institution, at room temperature (22 °C), under a 12 h light/dark cycle.

### 2.2. Sample Collection and Storage

At the end of the eight weeks, animals were induced anesthesia with ketamine 90 mg/kg and 10 mg/kg xylazine. Then, a midline incision was made and blood was obtained from the cava vein. After blood withdrawing, the heart and kidney were extracted. 

### 2.3. Plasma Biochemical Measurements

For plasma biochemical measurements, we used a brain natriuretic peptide (BNP) Kit ELISA mouse (Elabscience Biotech Co., Ltd., Wuhan, China). Plasma glucose was determined using a kit from Valtek (Santiago, Chile). Insulin was determined using an ELISA from EMD Millipore, Billerica, MA, U.S.A.

### 2.4. Histological Analyses

Cardiac and renal sections were obtained for pathology analysis. For this, hearts and kidneys were fixed in Bouin solution. Then, pieces of the organs were dissected, dehydrated in alcohol-xylol solutions and included in Paraplast. In a microtome, 5 µm sections were obtained and mounted in 0.1% polylysine-treated slides. After this, sections were rehydrated and prepared for hematoxylin–eosin, Masson’s trichrome, and periodic acid Schiff’s staining.

Glomerular pathological analysis was performed by a blinded investigator, scoring the degree of fibrosis, glomerular hypertrophy, and mesangial expansion according to previous reports [21,22,23,24].

### 2.5. TUNEL Assay 

Cardiac sections were probed with a Click-iT™ TUNEL Colorimetric IHC Detection Kit (Catalogue N° C10625, Thermo Fisher Scientific Inc., Carlsbad, CA, USA), for detection of apoptotic cells, as previously described [25,26].

### 2.6. Confocal Microscopy

Cardiac and renal sections were prepared for confocal microscopy, as previously described [27]. Renal sections were stained with anti-α-smooth muscle actin or F4/80 (Santa Cruz Biotechnology, Dallas, Texas, USA), followed by fluorescein isothiocyanate conjugated (FITC) antimouse (Jackson Inmunoresearch, West Grove, PA, USA). Nuclei were counterstained with propidium iodine (100 µM). Cardiac sections were probed for F4/80 to detect macrophages. Images were obtained with an LSM700 confocal microscope (Carl Zeiss, Jena, Germany).

### 2.7. Tetrahydrobiopterin (BH_4_) Quantification

Plasma and cardiac BH_4_ were determined as previously described [25,28] using a differential oxidation of biopterins protocol. Briefly, samples were submitted either to acidic or basic conditions (pH 3 or 9). Then, samples were oxidized with iodine. BH_2_ and BH_4_ contents were quantified by HPLC using HPLC (Perkin Elmer series 200, Waltham, MA) with fluorescence detection with excitation at 350 nm and emission at 450 nm.

### 2.8. Western Blot

Cardiac proteins were prepared as previously described for Western blot analysis [29]. Cardiac homogenates (30 µg) were mixed with loading buffer and summited to SDS-PAGE in 7.5% gels. Then, proteins were electro-transferred to nitrocellulose membranes. After blocking with Tween-buffered saline solution supplemented with 5% nonfat milk, membranes were incubated overnight at 4 °C with specific antibodies antinitrotyrosine (Badrilla, Leeds, U.K.). For NOS, SDS-PAGE was performed in nonreducing conditions of the loading buffer, and electrophoresis was run with the chamber immersed in ice. After electro-blotting, nitrocellulose membranes were incubated with anti-NOS1 antibody (Cell Signaling, Danvers, MA, USA) or NOS3 (BD Biosciences, Franklin Lakes, NJ, USA).

### 2.9. Statistical Analysis

Data are presented as means ± SEM, compared using ANOVA (normally distributed data) or Kruskal–Wallis test (nonparametric data) with Tukey’s or Dunn’s post hoc tests for comparisons between groups. A value of *p* < 0.05 was considered statistically significant.

## 3. Results

Three groups of mice were used: a group of normoglycemic mice, a group of streptozotocin-induced diabetic mice, and a third group of diabetic mice that received sapropterin (BH_4_) in drinking water, for eight weeks. At the end of the experimental period, mice were euthanized, and blood and organs were collected. Morphometric and blood parameters are presented in Table 1. These data confirmed the presence of hyperglycemia and reduced insulin levels in streptozotocin-treated mice. Additionally, cardiac hypertrophy was appreciated. Of these parameters, only cardiac hypertrophy was prevented by sapropterin treatment.

**Figure 1:**
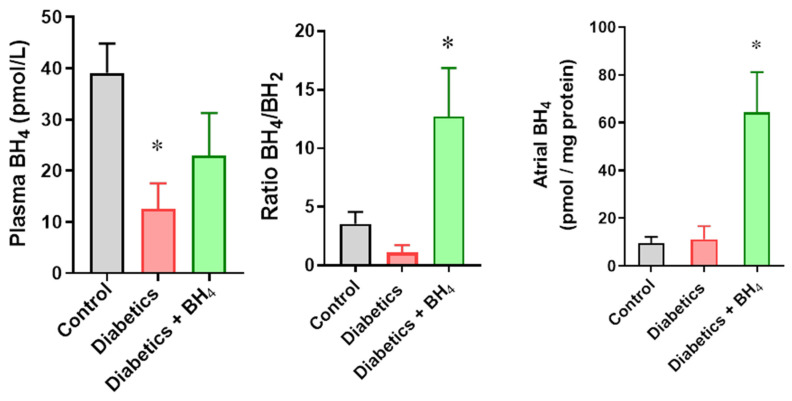
Oral administration of sapropterin restores levels of tetrahydrobiopterin (BH_4_). Left, plasma levels of BH_4_ in control, diabetics, and diabetic mice that received sapropterin (BH_4_) in drinking water. Right, intracardiac BH_4_ levels in control (black), diabetic mice (red), and diabetic mice that received BH_4_ (green). *n* = 6 in each group. * *p* < 0.05 vs. control and diabetics.

Next, we evaluated plasma levels of BH_4_ (Figure 1)_._ These were reduced in diabetic mice compared with those in normoglycemic controls, and increased toward normal in mice treated with sapropterin (39.1 ± 5.7 control, 12.5 ± 5 diabetic mice, and 22.9 ± 8.4 pmol/L in diabetic mice treated with BH_4_, *p* < 0.05), as well as the ratio between BH_4_ and its oxidized form BH_2_: 3.6 ± 1 control, 1.1 ± 0.6 diabetic mice, and 12.7 ± 4.1 in diabetic mice treated with BH_4_, *p* < 0.05 diabetic mice vs. diabetic mice + BH_4_. Intracardiac (intra-atrial) levels of BH_4_ were significantly increased in sapropterin-treated mice (8.8 ± 2.2 control, 9.6 ± 4.7 diabetic mice, and 209.7 ± 99.9 pmol BH_4_ mg/protein in diabetic mice + BH_4_, *p* = 0.0019).

**Figure 2:**
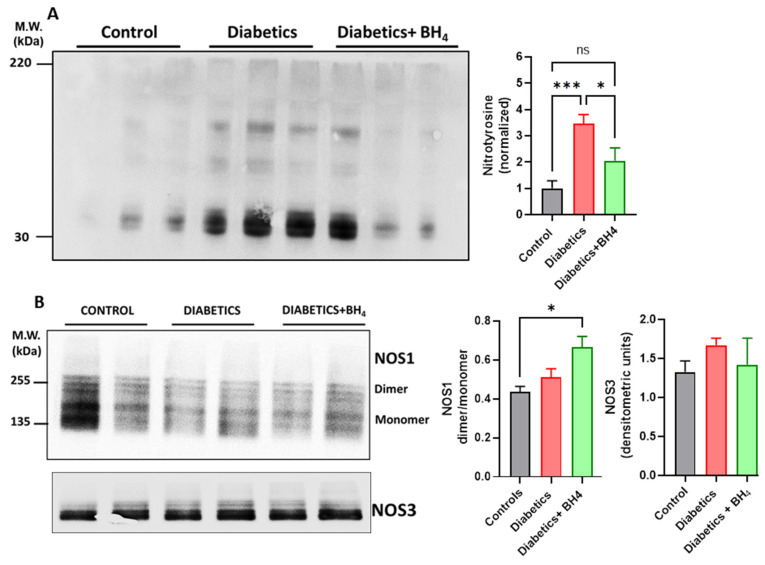
Oral administration of sapropterin reduces the intracardiac levels of oxidative stress. (**A**) Western blot analysis of nitrotyrosine in cardiac protein extracts from control, diabetic mice, and diabetic mice that received sapropterin (BH_4_) in drinking water. (**B**) Western blot for the levels of NOS1 and NOS3 in homogenates from control, diabetics and diabetics mice that received sapropterin (BH4) in drinking water. * *p* < 0.05, ***, *p* < 0.001. ns = not significant.

Next, we evaluated the impact of sapropterin treatment on cardiac oxidative stress (Figure 2). For this, we evaluated the levels of nitrotyrosine on intracardiac proteins by Western blot. This assay showed a significant increase in the content of nitrated proteins in diabetic mice compared with that of normoglycemic controls (*p* < 0.001), and this content was reduced in sapropterin-treated mice. In addition, we evaluated the levels of NOS1 presented as the dimer or monomer. Under reduced levels of BH_4_, NOS was unable to stabilize as a dimer and, as a consequence, was found in its monomeric form. Using SDS-PAGE under nonreducing conditions, the forms could be appreciated by Western blotting. This analysis showed that in our conditions, both the monomer and dimer of NOS1 were present. The NOS1 dimer and monomer levels were similar in the control and diabetic hearts. Nevertheless, the treatment with sapropterin increased the levels of dimer to monomer in diabetic hearts (*p* < 0.05). In the case of NOS3, the presence of the monomer was almost indistinguishable from that of the dimer. Neither of these constitutively expressed NOS showed changes in their expression levels (*p* > 0.05). These data suggested that sapropterin treatment was able to reduce intracardiac oxidative stress, probably independent of changes in NOS1 activity.

### 3.1. Cardiac Remodeling

**Figure 3:**
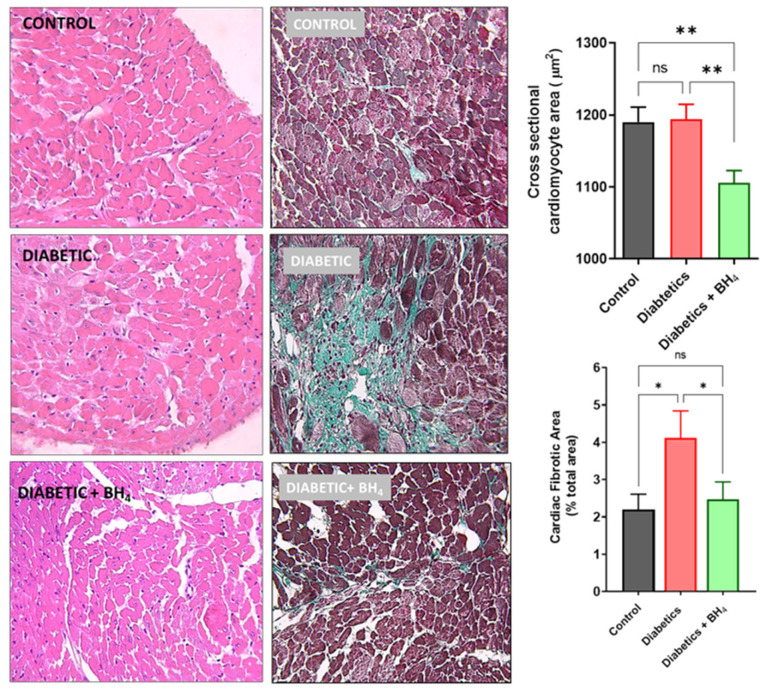
Cardiac remodeling in diabetes is ameliorated by oral administration of BH_4_. Left panel, representative hematoxylin and eosin stained cross-sections of hearts from control, diabetic, and diabetic mice that received BH_4_. Middle panel, representative Masson´s trichrome staining for collagen. Right panel, bar graphs depicting cardiac hypertrophy and fibrosis in control (black), diabetic mice (red), and diabetic mice treated with BH_4_ (green). * *p* < 0.05; ** *p* < 0.005 vs. the other groups. ns = not significant.

Diabetes induced cardiac hypertrophy, evaluated as the ratio of heart weight/tibia length, which was prevented in the group that received BH_4_: (7.6 ± 1.03 g/mm control, 8.6 ± 0.63 g/mm diabetic mice, and 7.38 ± 0.5 g/mm diabetic mice + BH_4_, *p* < 0.05, Table 1). In addition, hypertrophy was evaluated as cardiomyocyte cross-sectional area (Figure 3). This area was reduced in diabetic mice that received BH_4_ (1190 ± 460 µm^2^ control, 1194 ± 389 µm^2^ diabetic mice, and 1106 ± 375 µm^2^ diabetic mice + BH_4_, *p* = 0.0012). In addition, we evaluated cardiac fibrosis, which is also a hallmark of diabetic cardiomyopathy, by Masson’s trichrome staining. Diabetes induced cardiac interstitial fibrosis, which was reduced in mice that received BH_4_ treatment (2.2 ± 1.1% control, 4.12 ± 1.6% in diabetic mice, and 2.16 ± 1.2% in diabetic mice + BH_4_, *p* < 0.05). 

### 3.2. Apoptosis and Inflammatory Cells

**Figure 4:**
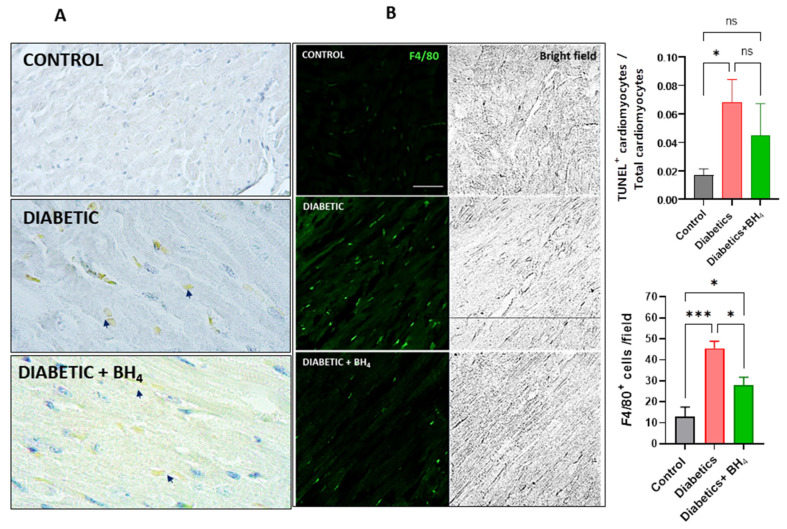
Impact of sapropterin in macrophage infiltration and cardiac apoptosis in diabetic mice. (**A**), representative images of TUNEL positive cells in cardiac sections obtained from control, diabetic and diabetic mice that received BH_4_. Arrows indicate TUNEL positive nuclei. (**B**), representative confocal microscopy immunofluorescence images of F4/80 (green) in cardiac sections from control, diabetic and diabetic mice treated with sapropterin (BH_4_). Asterisk * *p* < 0.05, *** *p* < 0.001 compared to the group indicated by brackets. ANOVA followed by Tukey *post hoc* test. Scale bar indicates 30 µm. ns = not significant.

Because there is significant cardiac damage in chronic diabetes, both apoptosis and the presence of inflammatory cells has been described in the diabetic myocardium. Apoptosis was evaluated as the presence of TUNEL-positive cells in cardiac sections (Figure 4A). Diabetes induced an increase in the percentage of TUNEL^+^ cardiomyocytes compared with normoglycemic hearts, but this increase was not modified in the hearts from sapropterin-treated mice. We also evaluated the presence of infiltrative inflammatory cells by immunofluorescence of F4/80, a cell surface marker that is present in macrophages (Figure 4B). Diabetic hearts showed an increase in the number of macrophages present in the myocardium compared with normoglycemic controls. This number was significantly decreased in the hearts of sapropterin-treated mice.

### 3.3. Renal Changes

**Figure 5:**
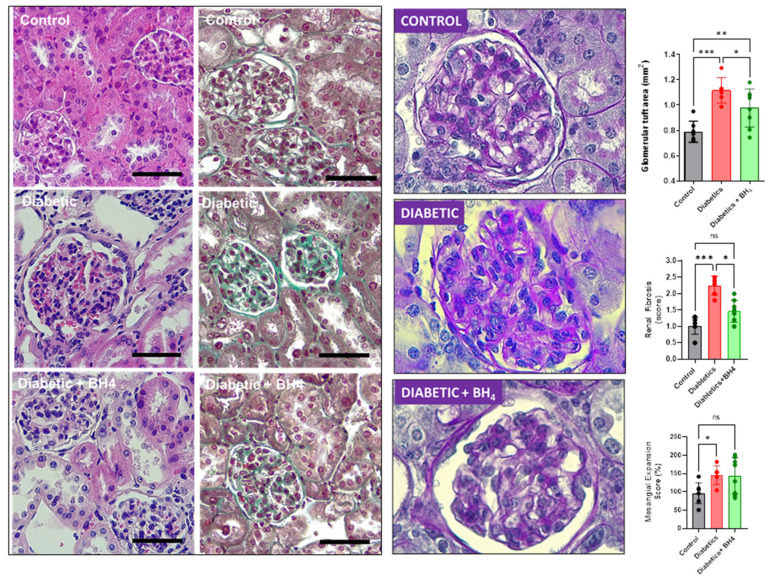
Renal morphological changes of diabetes are ameliorated by oral administration of BH_4_. Left panel, representative images of hematoxylin–eosin staining in renal sections from kidneys of control, diabetic, and diabetic mice that received BH_4_. Middle panel, representative Masson’s trichrome staining for collagen IV. Right panel, periodic acid-Schiff (PAS)-stained renal sections from kidneys of control, diabetic, and diabetic mice that received BH4. * *p* < 0.05; ** *p* < 0.01; *** *p* < 0.001 vs. the groups indicated by brackets. Bar indicates 50 µm. ns = not significant.

Next, we evaluated the impact of sapropterin treatment on diabetic nephropathy, evaluating glomerular morphology (Figure 5).

Diabetes induced glomerular hypertrophy compared to normoglycemic mice and was prevented by BH_4_ treatment (0.79 ± 0.08 mm^2^ in control, 1.12 ± 0.1 in diabetic and 0.98 ± 0.15 mm^2^ glomerular tuft size in diabetics + BH_4_, *p* = 0.0004). 

In addition, diabetic mice presented glomerular fibrosis, evaluated by Masson’s trichrome staining, which was prevented in mice that received BH_4_: 1.01 ± 0.25 in control, 2.25 ± 0.29 in diabetics and 1.46 ± 0.33 score units in diabetics + BH_4_ (*p* < 0.0001). Next, we evaluated mesangial expansion, which was increased in diabetic mice compared to controls, but was not reduced by sapropterin treatment (96.1 ± 10.7% control, 145.7 ± 10.4% diabetics, 143.5 ± 17.6% diabetics treated with sapropterin, *p* < 0.05 diabetics vs. control).

**Figure 6:**
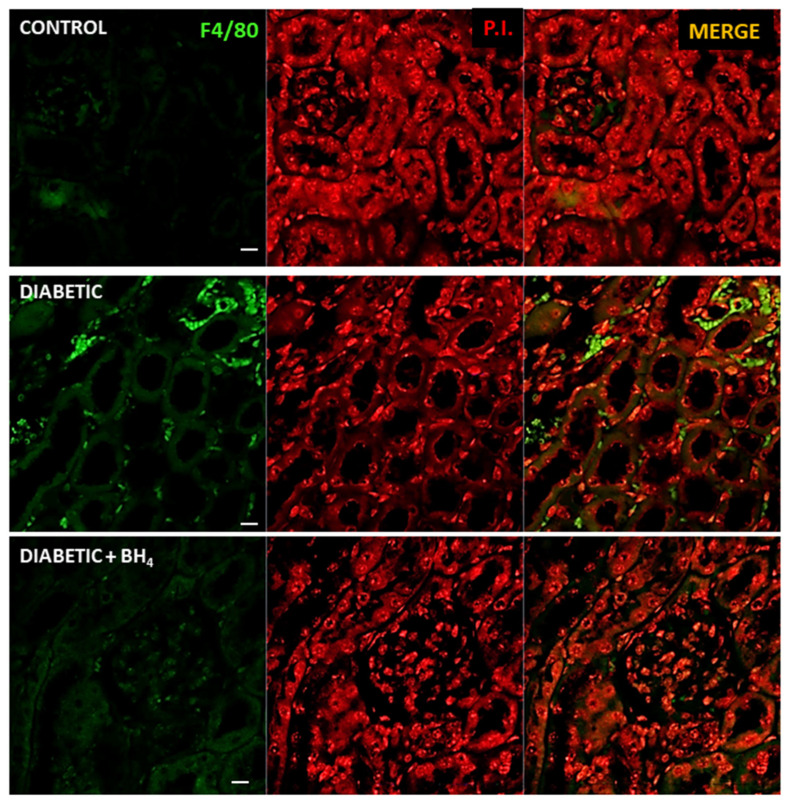
Renal tubular macrophage infiltration is prevented by oral administration of BH_4_ to diabetic mice. Representative confocal images of renal sections probed for F4/80 (green), a marker of macrophages, in normoglycemic control, diabetic, and diabetic mice treated with sapropterin (BH_4_). Middle panel, corresponding section counterstained with propidium iodide (P.I., red) for nuclei. Right panel, merge of both F4/80 and P.I: signals. Images were obtained at 40× magnification. Scale bar indicates 10 µm.

Next, we evaluated the degree of macrophage infiltration and the expression of α-smooth muscle actin. Macrophage infiltration was evaluated by immunofluorescence staining of the cell surface marker F4/80 (Figure 6). Renal sections of control normoglycemic mice did not show the presence of infiltrating inflammatory cells, neither in the glomeruli nor in the peritubular interstitium. On the contrary, F4/80-positive cells were extensively found in the peritubular space of diabetic mice. This infiltration was dramatically reduced in the diabetic kidneys from mice treated with sapropterin.

**Figure 7:**
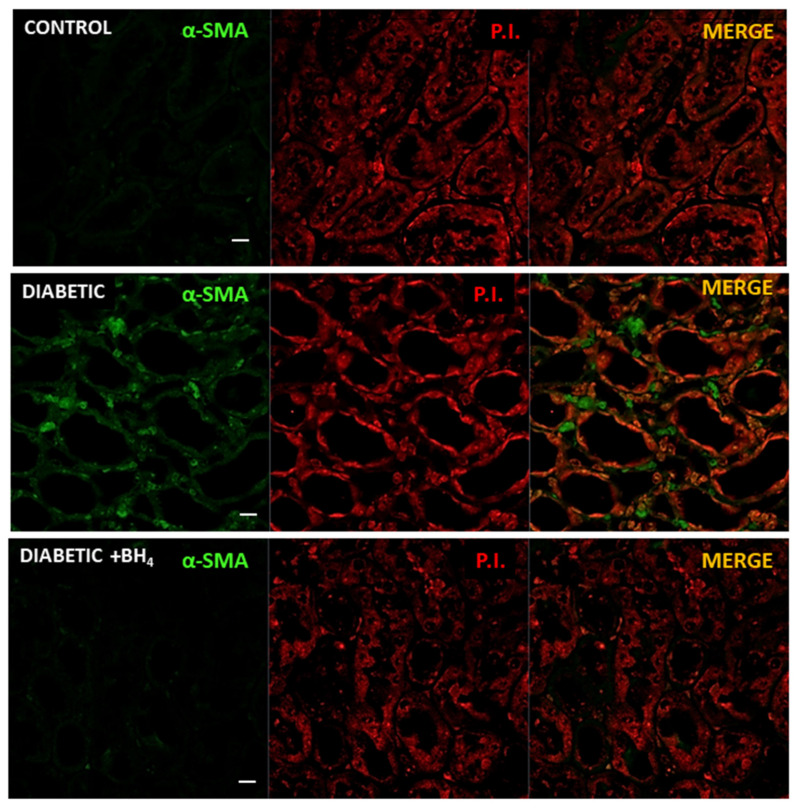
Renal tubular fibrosis was prevented by oral administration of BH_4_ to diabetic mice. Representative confocal images of renal sections (medulla) enriched in tubules probed for alpha smooth muscle actin (α-SMA, green), a marker of myofibroblast, in normoglycemic control, diabetic, and diabetic mice treated with sapropterin (BH_4_). Middle panel, corresponding section counterstained with propidium iodide (P.I., red) for nuclei. Right panel, merge of both α-SMA and P.I: signals. Images were obtained at 40× magnification. Scale bar indicates 10 µm.

In addition, we evaluated the presence of myofibroblasts as a marker of initial fibrosis (Figure 7). For this, renal sections were analyzed for α-smooth muscle actin (α-SMA). As expected, in the kidneys from normoglycemic control mice, there was no evidence of the presence of myofibroblasts in the peritubular space. On the contrary, the renal sections of diabetic mice showed the presence of these cells in the peritubular space mainly. Kidneys from sapropterin-treated mice showed almost no signal for α-SMA.

These results suggested that sapropterin treatment reduced macrophage infiltration and peritubular fibrosis in the diabetic kidneys. Overall, these results suggested that chronic treatment with BH_4_ in mice ameliorates the cardiorenal effects of diabetes_,_ probably by restoring the nitric oxide production. This offers a possible new alternative to explore a BH_4_-based treatment for the organ damage of diabetes.

## 4. Discussion

Our present results showed that oral administration of sapropterin (BH_4_) for one month to diabetic mice was able to ameliorate some pathological changes in both the heart and kidneys, two of the main organ targets of diabetes. Notably, BH_4_ administration reduced cardiac hypertrophy and fibrosis, while preventing glomerular hypertrophy in the kidney. These effects were associated with the reduction in oxidative stress, but apparently independent of NOS1 recoupling. Previous reports have shown that eNOS uncoupling is an important source of ROS in the diabetic kidney [30,31,32,33,34]. 

Reduced levels of tetrahydrobiopterin were attributed to the reduced expression of guanosine5′-triphosphate cyclohydrolase I (GTPCH), a rate-limiting enzyme in the synthesis of BH_4_ [35]. Experiments where GTPCH was overexpressed then reverted the phenotypes associated with diabetic nephropathy [32] and cardiomyopathy [36,37].

In the diabetic heart, NOS uncoupling has emerged as an important source of ROS [36,37,38,39], in a way that appears as an important therapeutic target to prevent the development of diabetic cardiomyopathy. Our results agree with those obtained by recent studies regarding the role of NOS1 uncoupling in the heart, which indicate that BH_4_ supplementation or genetic modifications that lead to the increased intracellular production of BH_4_ has a beneficial impact on the left ventricular function of diabetic mice. This effect is achieved by improving intracellular calcium handling, hence left ventricular systolic and diastolic mechanics. Interestingly, some of the positive effects of BH_4_ supplementation in the diabetic heart appear to be independent of NOS recoupling [39]. Our results are consistent with those findings because we did not observe significant recoupling of NOS1 in the diabetic heart after BH_4_ treatment, although we observed reduction in cardiac oxidative stress. It was suggested that this protective effect may be exerted by a metabolic action of NOS1, increasing the expression of insulin-independent glucose transporters (GLUT-1), which improved myocardial energetics [39]. In addition, it was also shown that BH_4_ exerts its beneficial effects in diabetic cardiomyopathy by activating peroxisome proliferator-activated receptor-γ coactivator 1-α (PGC-1α) signaling by interacting with calcium/calmodulin-dependent protein kinase kinase 2 (CaMKK2). These effects are also independent of NOS1 activity [40].

It was described in a model of cardiac hypertrophy by transverse aortic constriction that BH_4_ supplementation inhibits macrophage infiltration in the myocardium, probably by reducing the inflammatory signaling [41]. Interestingly, this protection is also independent of NOS uncoupling, which is consistent with a role of BH_4_ in macrophages biology. The exact mechanism through which BH_4_ mediates these anti-inflammatory effects remains to be determined, but it has been reported that BH_4_ is important for the macrophage functions, dependent and independent of iNOS [42,43]. 

We also observed a reduction in the number of macrophages present in the diabetic myocardium after treatment with BH_4_. Nevertheless, we did not evaluate the origin of these macrophages. The recent literature indicates the presence of at least four types of macrophages in the heart, with one being the infiltrating-monocytes-derived macrophages [44,45,46]. Particularly, a study using streptozotocin-induced diabetes in mice showed that cardiac macrophages producing interleukin 1β play an important role in the generation of arrhythmias in the diabetic heart [47]. A recent study documented that BH_4_ deficiency in macrophages increased the production of interleukin 1β and the inflammatory profile of these cells [48].

In the context of the diabetic patient, it is relevant to consider both the development of cardiomyopathy and nephropathy. Here, we showed that BH_4_ treatment was able to prevent the cardiac damage associated with the initial stages of diabetic cardiomyopathy, reducing macrophage infiltration, fibrosis, and hypertrophy, and similar effects were observed in the kidney at the glomerular and tubular levels. Interestingly, even though we observed a general beneficial effect of BH_4_ supplementation on renal morphology in diabetic mice, BH_4_ mesangial expansion was not modified. This is consistent with a previous report that suggested that BH_4_ may induce mesangial proliferation [49]. Plasma BH_4_ concentration was postulated as a predictor of renal function in diabetic patients [50].

Fibrosis has been identified as a common factor in cardiorenal syndrome [51,52], which is a reciprocal interaction between cardiac and renal dysfunction in several pathological states, including diabetes [53,54]. Here we verified that fibrosis affected both organs and was prevented by BH_4_ administration.

Importantly, sapropterin, a form of BH_4_, is available and approved for use in humans in phenylketonuric patients [55]. This opens the possibility that this drug may be considered for use in diabetes clinical trials. 

## 5. Conclusions

The results of the present study indicated that chronic oral administration of sapropterin (BH_4_) in mice ameliorates the morphological changes produced by diabetes in the heart and the kidney, probably by reducing oxidative stress, reducing inflammation, and the fibrotic processes that occur in the myocardium and at the glomerular and peritubular space in the kidney. This offers a possible new alternative to explore a BH_4_-based treatment for the organ damage caused by diabetes.

## Figures and Tables

**Table 1:** Morphometric and plasmatic parameters of control, diabetic and diabetic mice treated with tetrahydrobiopterin (BH_4_). BNP; brain natriuretic peptide. ANOVA followed by Tukey as *post-hoc* test. * *p* < 0.05 vs. control, ** *p* < 0.005 vs. control.

	Control	Diabetics	Diabetics + BH4	*p* Value
n	9	9	10	
Body weight (g)	40.7 ± 1.3	36.8 ± 1.0	34.0 ± 1.3 *	0.0026
Heart weight (g)	0.157 ± 0.010	0.161 ± 0.007	0.139 ± 0.003	0.0616
Heart weight/tibia length (g/mm)	7.57 ± 0.39	8.59 ± 0.34 *	7.39 ± 0.16	0.0495
Insulin (ng/mL)	1.09 ± 0.30 *	0.29 ± 0.12	0.21 ± 0.08	0.0037
BNP (pg/mL)	227.9 ± 25.3 **	96.1 ±13.5	66.6 ± 12.6	0.0495
Glucose (mg/dL)	137 ± 7.7 *	316 ± 69.7	247.3 ± 29.7	0.0132

## Data Availability

Data is available upon request.
